# Facts about Gas Poisoning

**Published:** 1923-03

**Authors:** 


### Facts about Gas Poisoning.

Sir,—With reference to your Note last month on this subject, perhaps you will allow me to point out that, although every new coal-gas fatality brings yet louder protests from an anxious public, yet it is obvious that the facts are still not generally understood. Carbon monoxide, the poisonous constituent, has always formed a part of coal-gas since it was first introduced to the public service. The impression that the Companies have recently added dangerous quantities (in the form of water-gas) is totally erroneous. Also the much-maligned Gas Regulation Act of 1920 simply gave power to prescribe a limit for the poisonous constituents, if, after inquiry, it was thought necessary to do so.

There never has been a legal limit, so that the Act can in no way be blamed for the recent fatalities. Actually, after due consideration, the best authorities recently decided to adopt no definite limit but to keep close analytical watch upon the gas supplied. The figures for the last twenty years, which are readily available, show that during that period there has been no increase in carbon monoxide percentage which could, in itself, be described as dangerous to life. In almost every case where death has occurred, the leakage of gas into the room has been so great that a slight variation in the amount of poison per volume of the gas would have made no difference at all to the result.

The real danger, and, therefore, the correct line of defence also, depends upon the amount of attention devoted to pipes, mains and gas fittings generally. Another point to remember, when blaming the advent of "water-gas," is the fact that this is not more difficult to detect. The smell of water-gas is more penetrating than that of coal gas, not less so, as is popularly supposed. It has often been suggested that some particularly pungent substance should be added to gas, thus making it almost impossible to overlook leakage. There are, however, many arguments against this innovation apart from its inevitable local effect in the average gas-supplied household. The fact certainly appears to be that gas as at present supplied is not in itself a danger.

Sound and modern means of conveyance and proper care of every fitting employed are two points to which we must expect the Companies to attend. The public must render intelligent assistance by never neglecting to notify an escape however trivial it at first may be. Do not blame the gas itself; see that it does not leak.

